# Optimization and Characterization of the Friction Stir Welded Sheets of AA 5754-H111: Monitoring of the Quality of Joints with Thermographic Techniques

**DOI:** 10.3390/ma10101165

**Published:** 2017-10-11

**Authors:** Luigi Alberto Ciro De Filippis, Livia Maria Serio, Davide Palumbo, Rosa De Finis, Umberto Galietti

**Affiliations:** Department of Mechanics Mathematics and Management (DMMM), Politecnico di Bari, 70126 Bari, Italy; luigi.defilippis@poliba.it (L.A.C.D.F.); liviamaria.serio@poliba.it (L.M.S.); rosa.definis@poliba.it (R.D.F.); umberto.galietti@poliba.it (U.G.)

**Keywords:** Friction Stir Welding (FSW), aluminum alloy 5754-H111, butt joint, thermographic techniques, thermal behavior

## Abstract

Friction Stir Welding (FSW) is a solid-state welding process, based on frictional and stirring phenomena, that offers many advantages with respect to the traditional welding methods. However, several parameters can affect the quality of the produced joints. In this work, an experimental approach has been used for studying and optimizing the FSW process, applied on 5754-H111 aluminum plates. In particular, the thermal behavior of the material during the process has been investigated and two thermal indexes, the maximum temperature and the heating rate of the material, correlated to the frictional power input, were investigated for different process parameters (the travel and rotation tool speeds) configurations. Moreover, other techniques (micrographs, macrographs and destructive tensile tests) were carried out for supporting in a quantitative way the analysis of the quality of welded joints. The potential of thermographic technique has been demonstrated both for monitoring the FSW process and for predicting the quality of joints in terms of tensile strength.

## 1. Introduction

Aluminum alloys are super lightweight construction materials used in several engineering applications and in the automotive, shipbuilding, aerospace and railway industries. Some of the most interesting aluminum alloys in aircraft and car manufactures [1] are the non-heat-treatable aluminum-magnesium (Al-Mg) alloys (5xxx series). The use of traditional welding methods on these alloys causes stresses, splashes, pores, slags, and other defects. The application of the innovative and modern solid-state Friction Stir Welding (FSW) process, if compared with fusion welding processes, offers many advantages referring to the process quality such as no splash, no smoke, no gas and no oxidation shielding.

Friction Stir Welding is a process developed and patented by The Welding Institute (TWI Ltd., Cambridge, UK) in 1991 [2]. It is a solid-state welding method based on frictional and stirring phenomena. In this process, welding heat is produced by a rotating, non-consumable tool, with a specially designed pin and shoulder. Other advantages with respect to the traditional fusion welding are: no mechanical distortions, minimal Heat Affected Zone (HAZ), and excellent surface finish [3]. Also, the FSW process creates potential joints with high fatigue strength, low preparation and little post-weld dressing.

The tool geometry, the welding parameters, and the joint designs are the significant parameters affecting the material flow pattern and temperature distribution, by determining the micro-structural evolution [4].

Different works [5,6,7,8,9] show as the transverse and the rotational tool speeds are the FSW process parameters that most affect mechanical properties of the joints. These latter depend mostly on the grain size and the dislocation density due to plastic deformation and recrystallization phenomena that occurring during the welding [10,11,12,13,14,15].

Attallah et al. [15] studied four alloys: 5251-H34, 5083-H116, 5754-O and 2024-T351 and on these materials, testing different FSW parameters. These analyses have highlighted the relationship between the banding of constituent particles and “onion rings” formation in AA 5754 joints.

In the work of Kulekci et al. [16], the effects of tool pin diameter and tool rotation speed at a constant weld speed were investigated on fatigue properties of friction stir overlap welded AA 5754. Two other works [17,18] provide information on the influence of process parameters, on the tensile and the fatigue behavior of a FSW joints in a tailor-welded blank of AA 5754. However, the welding parameters were not disclosed by the authors.

The process parameters are strictly correlated with the thermal behavior of plates and then with the mechanical properties of joints [19,20]. However, in literature, there are only a few studies about the thermal monitoring of the FSW process.

Hwang et al. [21], carried out an experimental study of temperature distributions within the work piece during FSW of aluminum alloys while Zhu et al. [20] performed a numerical simulation of transient temperature and residual stresses in the FSW process of 304L stainless steel. Chao et al. [22], assessed the heat transfer in FSW both experimentally and numerically, while Schmidt et al. [23], developed an analytical model for heat generation in FSW.

Recently, Serio et al. [24,25,26] demonstrated how the absolute temperature cannot be used for monitoring the FSW process in non-stationary conditions since it is affected by environmental conditions and is influenced by the experimental set-up adopted for the tests. In particular, a more sensitive thermal parameter has been proposed for investigating the thermal behavior of the material, representing the heat generated during the process.

In this work, an experimental approach based on thermographic technique has been used for optimizing the FSW process through thermal indexes related to the frictional power input. This paper starts from a previous work [26] in which the potential of thermographic techniques for evaluating the stationarity of the FSW process was demonstrated. Other tests were carried out in order to detect the optimal region of process parameters in term of tensile strength. In this regard, the Response Surface Methodology (RSM) including 2^2^ full factorial design and the steepest ascent have been used for detecting this region.

Finally, a suitable model previously developed [26] has been used for predicting UTS values of joints. The model acquires as inputs thermal parameters obtained by the analysis of thermographic data.

## 2. Experimental Design and Determination of the Optimal Region

The Response Surface Methodology [27,28,29,30] has been used with the aim of optimizing the process parameters (travel and rotation tool speeds). The RSM focuses on studying of a mathematical relationship between parameters and the response of the studied system. A basic approach of response surface methodology uses a first-degree polynomial model which is easily estimated by factorial experiments (Equation (1)).

y = β_0_ + β_1_x_1_ + β_2_x_2_ + β_12_x_1_x_2_ (1)

where x is a generic variable of the proposed model.

**Figure 1:**
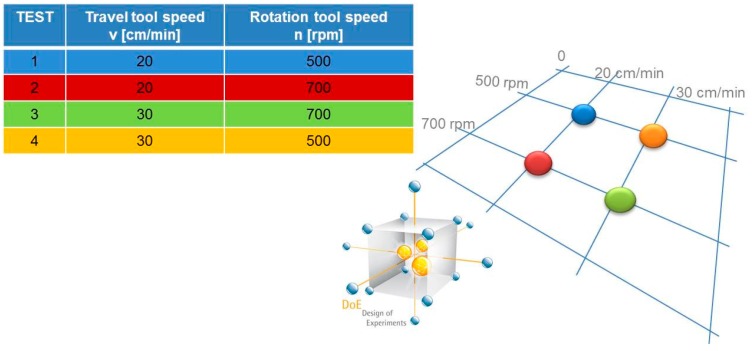
2^2^ full factorial plane.

The screening model, used for the first order situation, involves linear effects and a single cross product factor, which represents the linear interaction component. The initial screening has been carried out in previous works [24,25,26] by a 2^2^ full factorial experimental plan by considering the combinations of parameters shown in Figure 1.

The adopted values of the tool rotation speed and the travel speed were respectively: 500–700 [rpm] and 20–30 [cm/min]. A regression analysis has been obtained and a Screening Response Model has been defined, in which the chosen response variable “Y” is the Ultimate Tensile Strength (Equation (2)) [26]:

Screening Response Model: UTS = 597.31 − 15.942 v − 0.7763 n + 0.023985 n v(2)

where the UTS is expressed in [MPa], the rotation speed “n” [rpm] and the travel speed “v” in [cm/min]. Based on the fitted first-order model obtained from the 2^2^ full factorial screening plan, steepest ascent experiments were constituted. The direction of steepest ascent (descent) is the direction in which response increases (decreases) most rapidly. This direction is parallel to the normal line to the fitted response surface. Generally, the path of steepest ascent (descent) is chosen as the line through the center of the region of interest and normal to the fitted surface. Thus, the steps along the path are proportional to the regression first-order model 2^2^ full factorial screening plan (Equation (2)). The algorithm allows to determine the new experimental points, in which 2 factors were considered: x_1_ and x_2_. The linear model of the Y response becomes the following (Equation (3)):

Y = α_0_ + α_1_x_1_ + α_2_x_2_ (3)

where α_0_ is the known term of the model, and α_1_ and α_2_ are respectively the coefficients of the independent variables x_1_ and x_2_. In order to apply the steepest ascent method, it is necessary the normalization of the natural selected independent variables, considering as the origin points, the central point of the screening plan. Therefore, the independent variables correspond to the rotation and travel speeds (x_1_ = v; x_2_ = n), while the central point is identified by the combination v = 25 [cm/min] and n = 600 [rpm]. By normalizing the independent variables and applying a linear regression to the data expressed in these normalized variables, the following first-order model (Equation (4)) is obtained:

*Y* = 92.75 − 7.755 v_norm_ − 17.667 n_norm_ (4)

**Figure 2:**
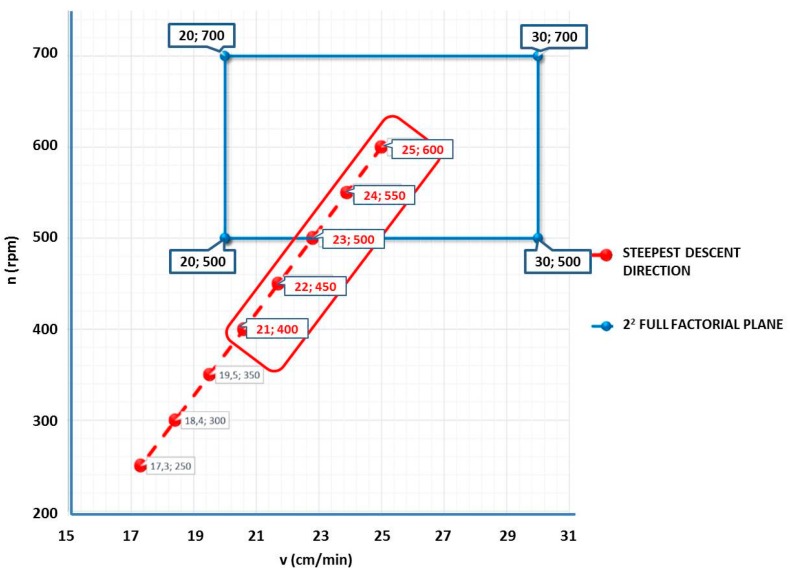
Full factorial plane and steepest descent direction definition.

In Figure 2 and in Table 1, the test sequence is shown. It has been extrapolated by the direction in which the response decreases most rapidly.

## 3. Materials and Methods

Single pass butt welds were produced in 6 mm thick plates of AA 5754-H111, with the following dimensions: 150 mm × 700 mm (width × length). This aluminum alloy is characterized by an excellent corrosion resistance in the marine environment and it exhibits high formability. It is widely used as coating of pressure vessels, tankers, chemical plants. All these applications make the alloy particularly useful for the manufacturing of components for automotive and naval fields. The chemical compositions and principal mechanical properties of the AA 5754-H111 are, respectively, presented in Table 2 and Table 3.

All welds were carried out in “position control” using a FSW machine (LEGIO™ FSW 4UT produced by ESAB, Laxå, Sweden). It is equipped with 4 axes and controlled using the latest of Programmable Logic Controller (PLC) technology.

**Figure 3:**
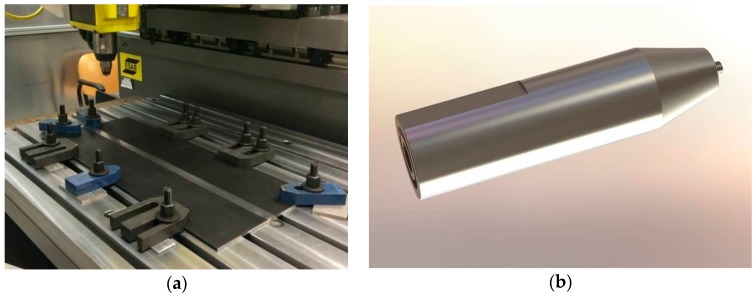
(**a**) Backing-plate and clamp for the rigid clamping of the pieces and (**b**) tool geometry used for the FSW tests.

The work pieces were fixed on a rigid backing-plate and clamped along the welding direction to avoid transversal movements during welding (Figure 3).

The FSW dwell time was always kept at 15 s, later the tool moves with constant welding speed according to the parameters combination shown in Table 1. During the penetration phase, the rotating tool pin penetrates into the work piece until the tool shoulder makes contact. The penetration speed is about 0.5 cm/min. The tool has a shoulder diameter of 22 mm, a height of the pin of 5.8 mm and a tilt angle of 1.2° to facilitate the mixing of the material (Figure 3).

### 3.1. Non-Destructive Tests

Visual inspections, macrographic and micrographic tests have been carried out preparing the cross-sectional samples taken from all welded joints. Specimens were prepared using standard metallographic methods for macroscopic examinations of the weld zones. The face examination of the welds was carried out according to the criterion fixed by the Standard UNI EN ISO 25239-5:2012 [31]. Cross sections of the welds were cold mounted, polished and etched with a Keller solution. The joints surface was examined with 50× and 200× magnification.

### 3.2. Thermographic Technique

**Figure 4:**
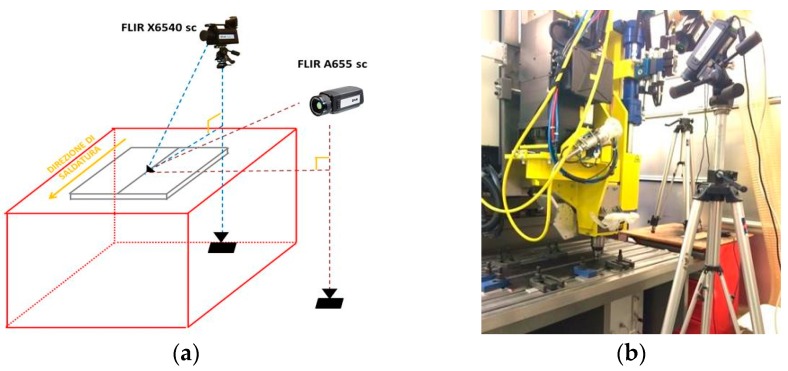
Set-up of the two IR cameras: schematic representation (**a**) and picture (**b**).

The surface thermal acquisitions were performed using two IR-cameras. In particular, in order to acquire thermal data along the weld tool direction, the cooled FLIR X6540 SC IR camera has been used. This latter has thermal sensitivity (NETD) <20 mK and is based on a InSb cooled detector with 640 × 512 pixels. The uncooled-microbolometric FLIR A655 SC IR camera has been placed in a perpendicular direction with respect to the first thermocamera (thermal sensitivity (NETD) <30 mK, 640 × 480 pixels) (Figure 4).

The infrared sequences were recorded during each test at frequency of 15 Hz. Both thermal cameras recorded the maps of surface temperature across the weld, for each FSW process parameter combination. Before the tests, all specimens were painted with matt black coating (see Figure 4) in order to make uniform the surface emissivity (0.95) and to avoid reflections caused by any heat sources close to the specimens during the tests. In the Section 4.5, the results of analysis carried out on thermal sequences obtained with the A655sc IR camera are shown.

### 3.3. Tensile Tests

Ultimate Tensile Strength was used to evaluate the mechanical properties of welded joints. All tests have been performed on a INSTRON Series IX 3360 (INSTRON, Norwood, MA, USA) under displacement control with a constant crosshead speed displacement rate of 5 mm/min according to Standard ISO 6892-1:2016 [32].

**Figure 5:**
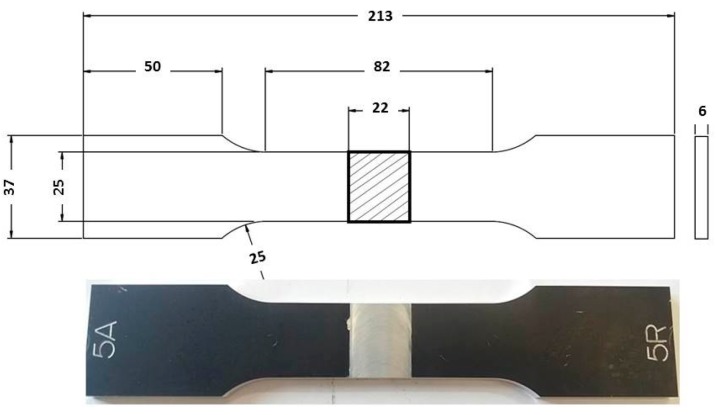
Specimen geometry.

Tensile tests were performed both on FSW joints and AA 5754-H111 base material, on total number of five samples. The specimens were designed in accordance with UNI EN ISO 4136:2012 standard [33]. The specimen geometry is shown in Figure 5.

## 4. Results and Discussion

### 4.1. Visual Inspection

**Figure 6:**
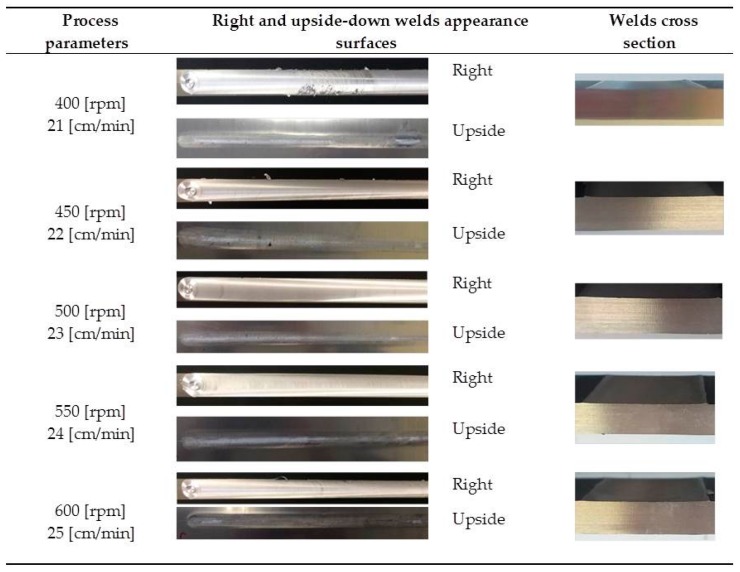
Right and upside surface of the FSW joints.

The FSW joints were manufactured according to the various process parameters combinations (Table 1) and their overall appearance is shown in Figure 6.

Interesting results can be obtained by visual inspection, due to the possibility of verifying the presence of possible macroscopic external defects, such as surface irregularities, excessive flash, and lack of penetration or surface-open tunnels. No significant cracks, voids, wormholes or other surface deformities were observed in any of the weld samples produced. All joints have little or no flash on either side of the junction line. The penetration appears to be complete through the thickness of the plates. The right and upside-down welds appearance surfaces are uniform and clean, free of ripples, indicating a complete mixing of the material.

The surface of all FSW joints is characterized by the presence of a series of slight cycloidal ripples on the surface, which are produced by the tool shoulder rotation. The distance between these ripples is determined by the ratio between the rotation tool speed and the travel tool speed (this measure generally increases with the travel tool speed).

Standard [33] does not define specific acceptability criteria for defects in FSW joints for the visual testing so, acceptability levels have been fixed (Table 4) to discern and quantify the presence of defects.

**Figure 7:**
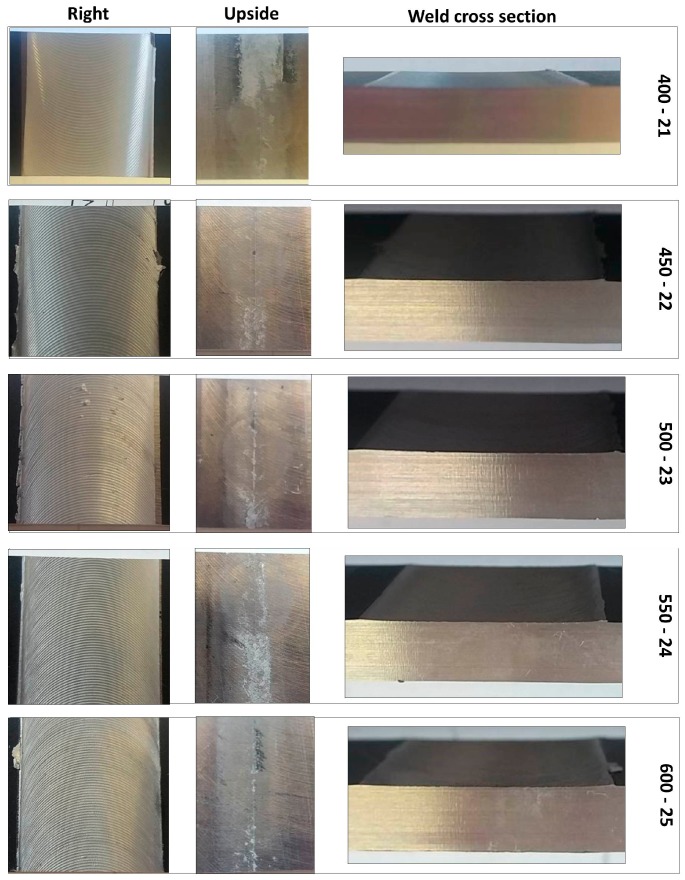
FSW aluminum alloy A 5754-111: visual inspection test reports.

According to the above criteria, the following figure (Figure 7) show the results of visual tests, which are performed for all FSW parameters.

Summarizing (see Table 4), it is possible to define that the global welds observation confirms the good surface finish; in fact, the flash production is minimal and no apparent surface defects are detected on any joint-for all joints there is full penetration and the “Slitting of the welded surface” and ripples on the surface are in the range of acceptability.

### 4.2. FSW Downward Forces

**Figure 8:**
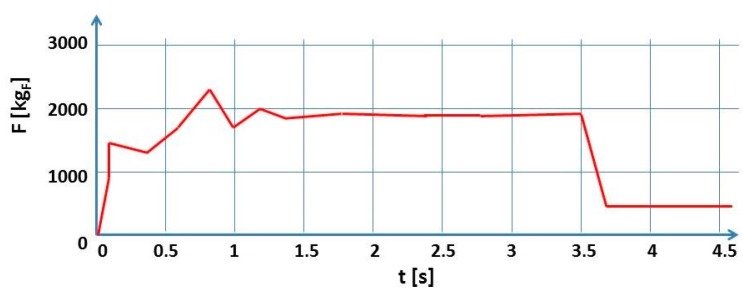
Downward FSW force trend for the test 500 [rpm]–23 [cm/min].

For all tests, the downward force F_z_ (the most significant contribution of the force components during the FSW process) has been monitored. As example, in Figure 8 is shown the F_z_ temporal profile in correspondence of a specific input parameter conditions: 500 [rpm] and 23 [cm/min].

The downward force varies directly with plunge depth. Therefore, it is possible to clearly highlight a first transitional section and a second steady section in which the trend of the downward force is constant. The downward force did not exceed 20 kN at any time during welding. In Sub-Section 4.6 the correlation with thermal profile will be discussed.

### 4.3. Macrostructures

**Figure 9:**
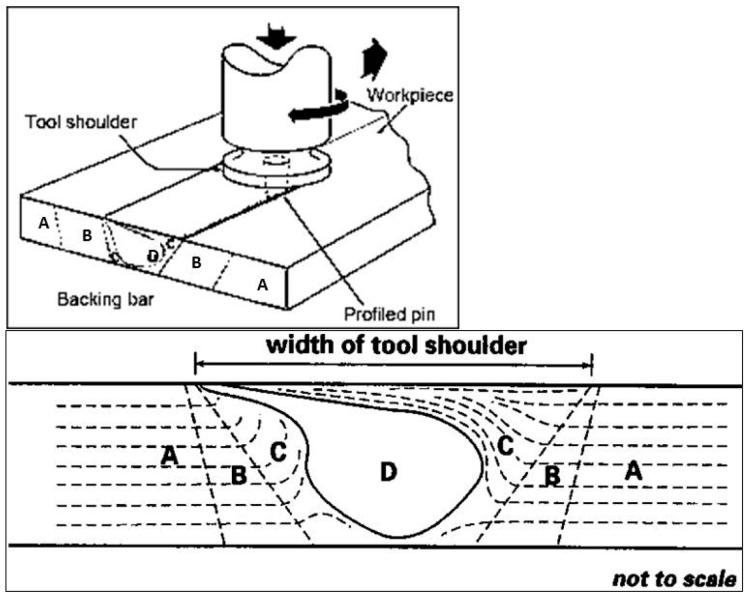
FSW joint characteristic areas: (A) Base material; (B) Heat Affected Zone; (C) Thermo-Mechanically affected zone—TMAZ; (D) Nugget. Friction stir welding principle and microstructure: (A) Base material; (B) Heat affected zone; (C) Thermomechanically affected zone (TMAZ); (D) Weld nugget [15,35].

**Figure 10:**
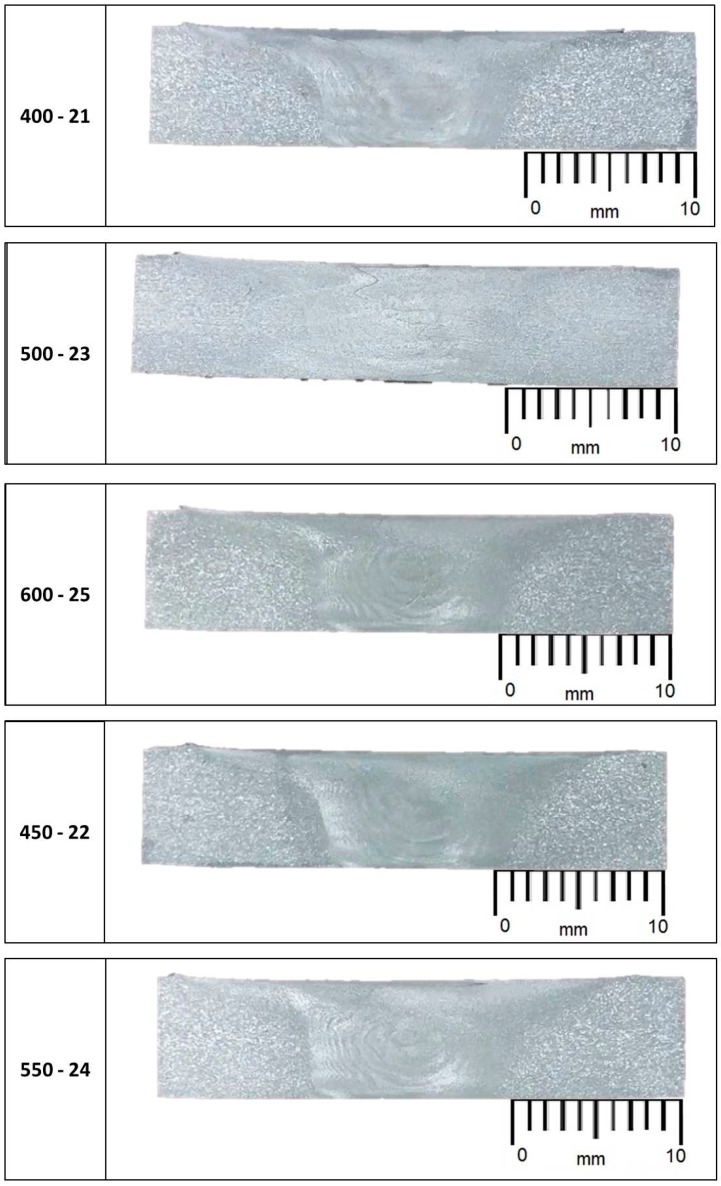
Macro structure of the FSW joints.

Four distinct microstructural zones i.e., stir zone, weld nugget, the thermomechanically affected zone, and heat affected zone are present in welded area (Figure 9). Figure 10 shows all macrostructures performed for all FSW process parameters. These latter were carried out in accordance with UNI EN 17637:2017 [34].

In the nugget, it is possible to observe the correct mixing of the welded material and the “onion ring structure”, typical of the FSW process. Furthermore, it is evidence of a correct tool penetration for all the joints. All specimens present an excess material under the tool shoulder which solidifies on the material surface (Figure 8).

### 4.4. Microstructures

**Figure 11:**
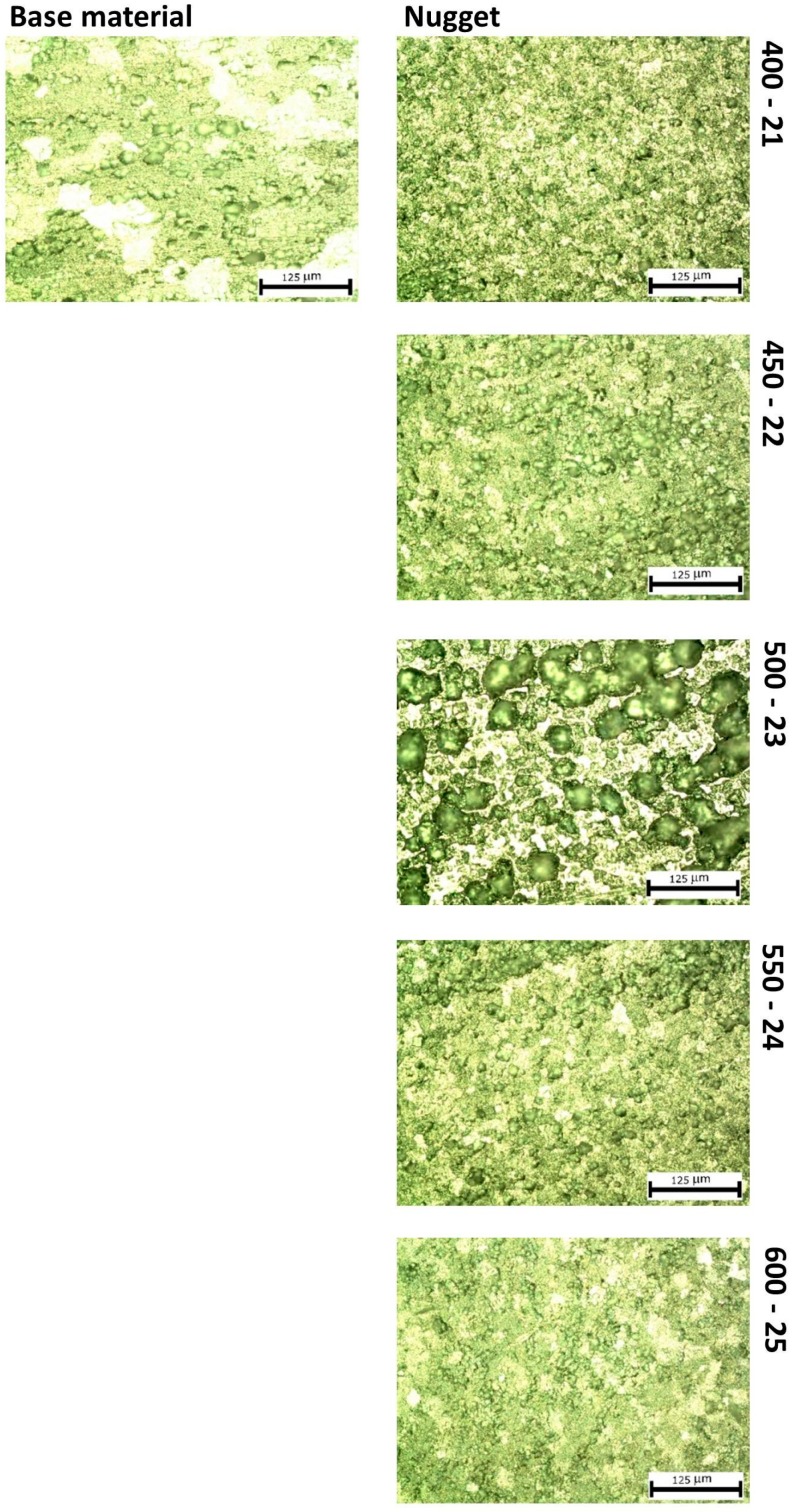
200× microstructures of the base material and nugget zones.

Figure 11 shows the microstructures of base material nugget zones obtained with 200× magnification. Despite some problems with the etching procedure in the nugget zone, it is possible to observe a refinement of the grain size with small, relatively equal grains due to the recrystallization action of the material by the tool.

**Figure 12:**
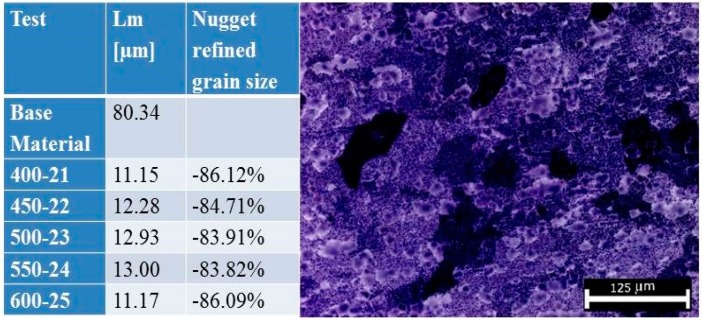
Grain size refinement of the nugget zone.

Figure 12 shows, also quantitatively, the significant crystal grains refinement observed (reduction of the grain size of approximately 85%) for all FSW joints with consequent improvement of the mechanical properties.

### 4.5. Thermal Behavior of Joints: Analysis of Thermographic Data

In this work, the thermographic technique has been used for monitoring the FSW process in order to predict and evaluate the quality of joints. To do this, two thermal indexes [24,25,26] have been used for describing the thermal behavior of the aluminum plates during tests: the maximum temperature (*T_max_*) and the maximum heating rate of material or maximum slope of heating curve (*MSHC*).

**Figure 13:**
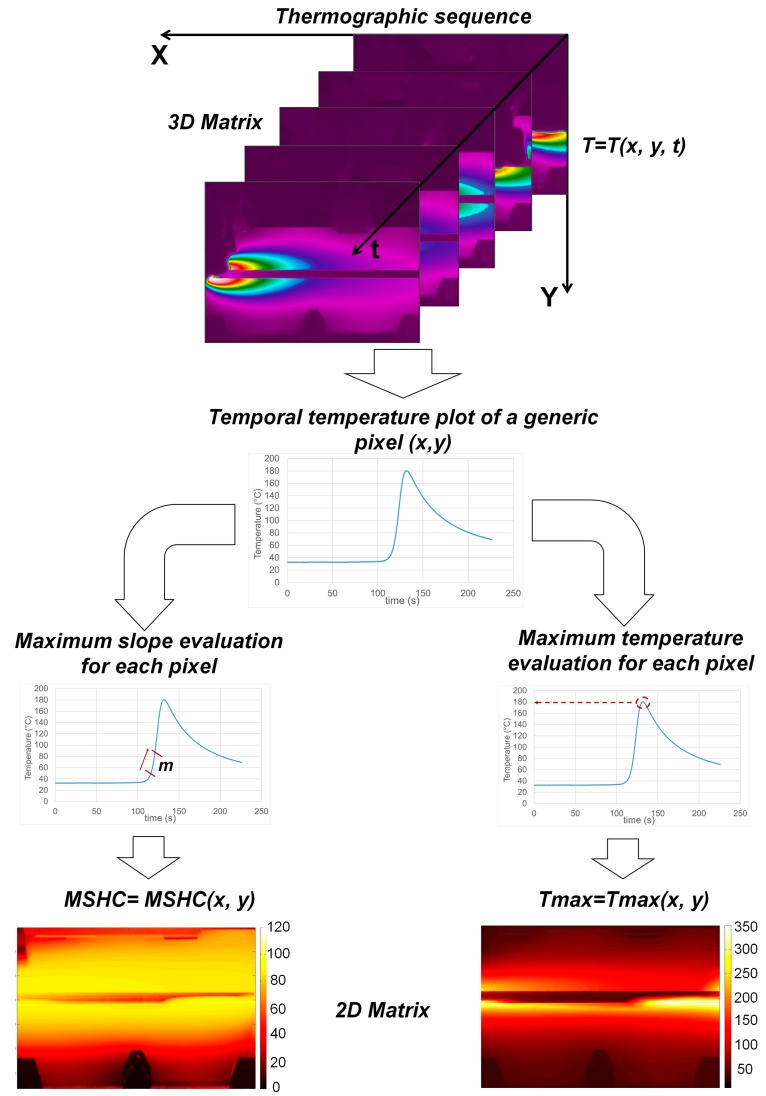
Scheme of the procedure used to obtain the maps of *T_max_* and *MSHC*.

Figure 13 provides detailed explanation of physical meaning of such the parameters and shows the algorithm used for the analysis of thermographic sequences, which consist of several steps:

1. Acquisition of the thermographic sequence representing welding process (to build 3D Matrix),
2. Extrapolation, pixel by pixel, the temporal temperature data *T = T*(*x*,*y*,*t*)
3. For each pixel, evaluation of the maximum temperature (*T_max_*)
4. For each pixel, evaluation of the maximum heating rate (*MHSC*). In this case, the maximum slope of the heating curve is assessed for m = 200 data,
5. Obtaining 2D maps related to the two indexes.

**Figure 14:**
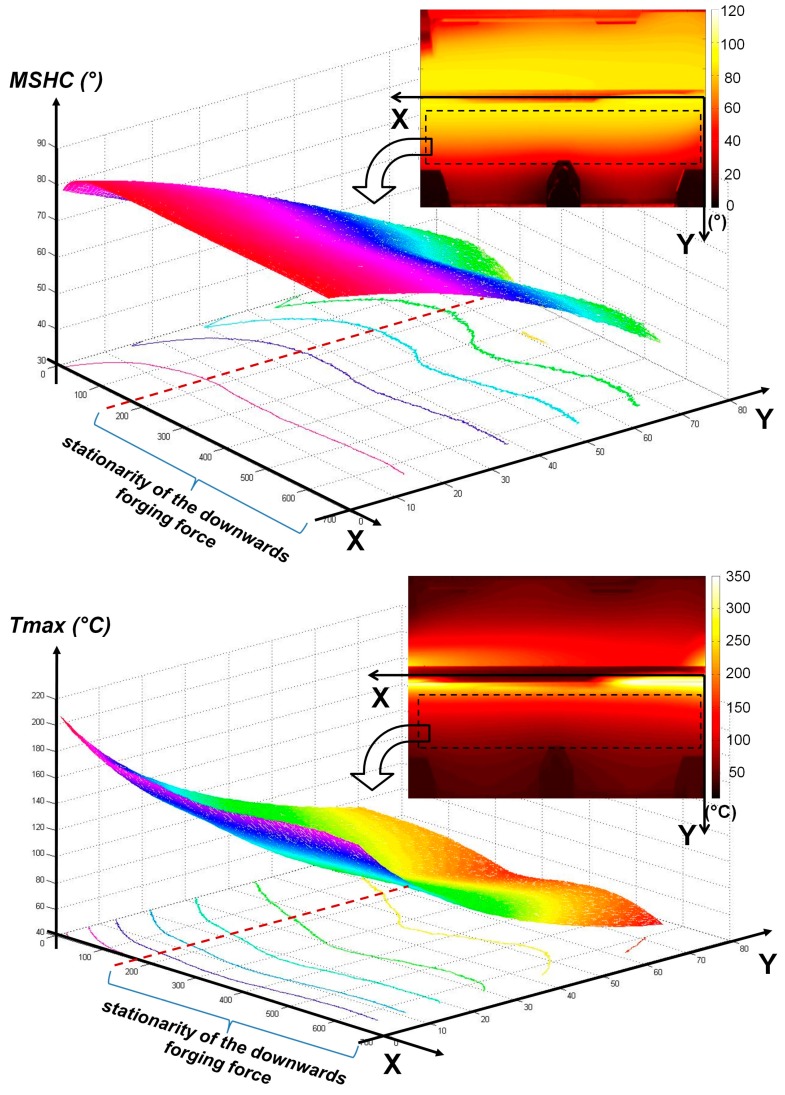
Maps of the thermal indexes and relative data profile along *X* and *Y*-axes (Test 3).

In Figure 14, the two maps related to the two thermal indexes and trends of some profiles along the *X*-axis are reported. It is very clear as, along the *X*-axis, both the indexes have a non-constant value due to the non-stationary conditions of the FSW process, in the first part of welding. In this regard, it is very interesting to observe that the downward force stabilizes its value at the same time as the thermal indexes. These results confirm the potential of the thermographic technique for monitoring the stationarity of the FSW process.

As it is shown in Figure 14, a variation of the shape of the profiles can be also observed along the *Y*-axis. In this case, profile variations are due to the presence of grips (see Figure 3) used to fix the position of the plates during the tests. More precisely, grips and the set-up used for tests influence in significant way the thermal behavior of plates and then thermal indexes values along the *Y*-axis.

**Figure 15:**
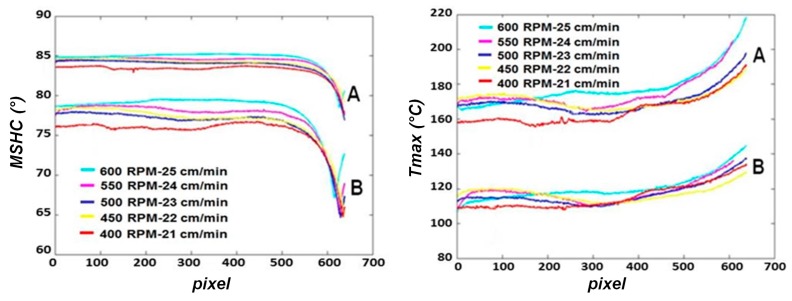
Indexes profiles along the *X*-axis and at two different distances from the center of the welding (A = 20 mm, B = 37 mm).

Figure 15 shows the profiles of both indexes for each test at respectively 21 mm (profile A) and 37 mm (profile B) from the welding. In particular, as demonstrated in the work of Serio et al. [24], *MHSC* values are more stable than maximum temperature for all tests. In fact, the *T_max_* is influenced more than the *MSHC* from heat exchanges for convection and conduction with environment and grips.

To evaluate the influence of each combination of process parameters on both indexes, a one-way ANOVA has been carried out by considering 5 equidistant data from each profile (A) in the stationary zone. It is interesting to underline as these results can be compared with those obtained in Serio’s work [21] since the same distance from the center of welding has been considered (y = 21 mm).

In Table 5 and Table 6 are reported all value of *T_max_* and *MSHC* for all tests while, Table 7 and Table 8 show as both proposed indexes are significant for the combination of process parameters chosen for tests.

### 4.6. Mechanical Properties of the FSW Joints and Relationships with the Thermal Indexes

All the specimens were machined according to standard UNI EN ISO 6892-1:2016 [33] and were obtained in orthogonal direction with respect to the rolling direction and extracted in the stationary region of downward force.

As shown in Table 9 the failure of joints occurs within the gauge length, far away from the weld zone.

The above data (Table 10) show an increase of the UTS of the FSW joints in comparison with the mechanical properties of the base material. In addition, for all tests a mechanical properties recovery of about 5% has been recorded with respect to the properties of the base alloy. This demonstrates the high quality of welds obtained in this experiment.

In literature [36], it has been demonstrated as the UTS is directly correlated to the frictional power input (FPI) expressed as:

(5) $$P_{\mathrm{in}}\;=\;\frac{4}{3}\pi \mu F_{z}\omega r$$

where *μ* is an effective coefficient of friction under the tool shoulder, *r* is the radius of the tool shoulder and *ω* is the rotation tool speed. In particular, higher values of UTS are obtained in correspondence of lower values of FPI. It is interesting to notice as in Serio’s work [26] a similar correlation has been verified between *MSHC* data and UTS. In fact, the heat generated during the FSW process is proportional to the FPI and in this regard, *MSHC* data can be considered as an index of the frictional power input.

**Figure 16:**
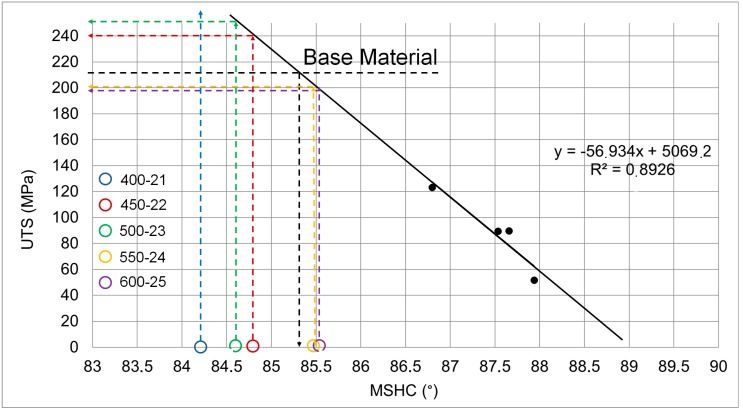
Empirical model proposed in [26] and relative predicted values for each test.

Figure 16 is shows the model reported in the work of Serio et al. [26]. Although this latter has been obtained for non-stationary conditions, it allows for predicting and estimating the UTS of joints. In fact, by considering the thermographic results obtained in this work, *MSHC* values < 85.35 deg provide UTS values above the base material ones (tests 1, 2, 3) while test 5 presents the higher value of *MSHC* that provides the lower value of UTS, in agreement with the results of tensile tests. Only for the test 4 the expected UTS value is lower than the experimental one. This error can be justified considering that the empirical model proposed in [26] has been obtained with few points and with different plate dimensions. However, the thermal index *MSHC* allows for detecting the optimum region of the FSW process in terms of strength of joints.

## 5. Conclusions

In this work, destructive and non-destructive tests were carried out to evaluate the quality of aluminum welded joints obtained with the FSW process. An experimental plan has been developed by considering travel and rotational tool speeds as process parameters in order to assess the optimum region of the process. The main results can be summarized as follows:

- Defect-free welds were observed from the visual inspection for all combinations of process parameters. Macrograph and micrograph inspections revealed a good mixing and a good penetration of the tool.
- Tensile tests have shown a recovery of about 5% of UTS with respect to the base material with a maximum value in correspondence of n = 500 rpm and v = 23 cm/min.
- The potentiality of thermography for the on-line monitoring of the FSW process was demonstrated along with the possibility to predict the quality of joints in terms of Ultimate Tensile Strength by monitoring thermal parameters.

Future works will be focused on the determination of more accurate empirical models to relate the thermal behavior with the strength of joints and to validate the proposed approach on different materials and joint configurations.

## Figures and Tables

**Table 1:** FSW Process parameters combinations according the steepest descent method.

Test	Step	n_norm_	v_norm_	n	v
1	Origin	0.0	0.0	600	25
2	+∆	−0.5	−0.2196	550	24
3	+2∆	−1.0	−0.4392	500	23
4	+3∆	−1.5	−0.6588	450	22
5	+4∆	−2.0	−0.8784	400	21

**Table 2:** Chemical composition of the AA 5754-H111.

Chemical Composition								
Si	Fe	Cu	Mn	Mg	Cr	Ni	Zn	Ti
0.40	0.40	0.10	0.50	2.60–3.60	0.30	0.05	0.20	0.15

**Table 3:** FSW mechanical and thermal properties of the AA 5754-H111.

Mechanical and Thermal Properties					
R_m_ (MPa)	Rp_0.2_ (MPa)	E (MPa)	Density (g/cm^3^)	Conductivity (W/m°C)	Specific heat (Cal/kg°C)
190	80	70000	2–65	138	0.213

**Table 4:** Acceptability criteria for visual testing conducted on FSW butt joints FSW aluminum alloys.

N. Criteria	Indicators	Acceptability Levels
I	Flash	Max. measure: 3 mm
II	Voids on the surface	Max. measure: 5 mm
III	Ripples	Max q.ty = 20% on the surface
IV	Penetration	Full
V	Slitting of the welded surface	Max. measure: 1 mm

**Table 5:** *T_max_* values obtained for each test at y = 21 mm.

Measured Points on Profile	Test				
	1	2	3	4	5
**1**	168.2	177.0	173.4	187.2	183.3
**2**	169.4	176.1	171.7	186.9	184.2
**3**	170.1	175.0	171.4	185.1	183.4
**4**	170.7	173.4	171.4	183.8	183.7
**5**	170.4	173.2	171.1	183.6	183.6
**Mean (°C)**	169.8	174.9	171.8	185.3	183.6
**Standard Deviation (°C)**	1.0	1.7	0.9	1.7	0.4

**Table 6:** *MSHC* values obtained for each test at y = 21 mm.

Measured Points on Profile	Test				
	1	2	3	4	5
**1**	84.2	84.9	84.7	85.6	85.5
**2**	84.2	84.8	84.7	85.5	85.7
**3**	84.2	84.8	84.6	85.5	85.6
**4**	84.2	84.7	84.6	85.5	85.6
**5**	84.2	84.7	84.6	85.5	85.6
**Mean (deg)**	84.2	84.8	84.6	85.5	85.6
**Standard Deviation (deg)**	0.03	0.08	0.06	0.04	0.02

**Table 7:** One-way ANOVA: *T_max_* vs. combination of process parameters.

Source	DF	SS	MS	F	P
FSW parameters configuration	4	984.85	246.21	163.14	0.000
Error	20	30.18	1.51	-	-
Total	24	1015.04	-	-	-
**Significance Level: 0.05**					
*S* = 1.228; *R* − *S*_q_ = 97.03%; *R* − *S*_q_ (adj) = 96.43%					

**Table 8:** One-way ANOVA: *MSHC* vs. combination of process parameters.

Source	DF	SS	MS	F	P
FSW parameters configuration	4	6.62	1.66	646.05	0.000
Error	20	0.05	0.00	-	-
Total	24	6.67	-	-	-
**Significance Level: 0.05**					
*S* = 0.051; *R* − *S*_q_ = 99.23%; *R* − *S*_q_ (adj) = 99.08%					

**Table 9:** Transverse tensile tests.

Positions Tensile Failures				
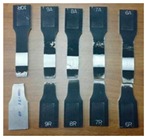	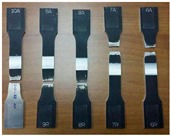	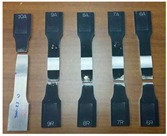	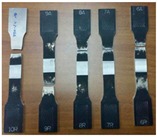	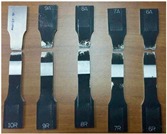
400 [rpm]	450 [rpm]	500 [rpm]	550 [rpm]	600 [rpm]
21 [cm/min]	22 [cm/min]	23 [cm/min]	24 [cm/min]	25 [cm/min]

**Table 10:** Tensile test results (UTS) and percent elongation (A%).

Process Parameters: n [rpm] v [cm/min]	UTS			A			%UTS_BM_	% Recovery of the Mechanical Properties
	Average Values [MPa]	Std. Dev. [MPa]	Std. Dev./Average	Average %	Std. Dev.	Std. Dev./Average		
Base material	210.07	3.11	-	-	-	-	-	-
400–21	220.09	0.51	0.00	16%	0.00	0.02	104.77%	+4.77%
							UTS_BM_	
450–22	220.05	2.09	0.00	17%	0.01	0.04	104.77%	+4.77%
							UTS_BM_	
500–23	221.91	1.31	0.01	17%	0.01	0.07	105.64%	+5.64%
							UTS_BM_	
550–24	221.21	3.46	0.02	18%	0.01	0.05	105.30%	+5.30%
							UTS_BM_	
600–24	204.55	18.15	0.09	17%	0.02	0.09	97.37%	−2.63%
							UTS_BM_	

## Abbreviations

The following abbreviations are used in this manuscript:

FSW	Friction Stir Welding
AA	Aluminum Alloy
UTS	Ultimate Tensile Strength
PLC	Programmable Logic Controller HAZ Heat Affected Zone
FSWP	Friction Stir Welding Process Parameters
Tp	Thermal Parameters
WP	Weld Pitch
TMAZ	Thermo-mechanically affected
MSHC	Maximum Slope of Heating Curve
FPI	Frictional Power Input

## References

[1] Shtrikman M.M. (2008). Current State and Development of Friction Stir Welding Part 3. Weld. Int..

[2] Thomas W.M., Nicholas E.D., Needham J.C., Murch M.G., Temple-Smith P., Dawes C.J. (1991). Friction Welding. The Welding Institute TWI 1991. Patent Application No..

[3] Rodrigues D.M., Leitao C., Lauro R., Gouveia H., Laureiro A. (2010). High speed friction stir welding of aluminium alloys. Sci. Technol. Weld. Join..

[4] Elangovan K., Balasubramanian V. (2007). Influences of pin profile and rotational speed of the tool on the formation of friction stir processing zone in AA2219 aluminium alloy. Mater. Sci. Eng. A.

[5] Jeom K.P. (2009). Mechanical properties of friction stir welded aluminum alloys 5083 and 5383. Int. J. Naval Archit. Ocean Eng..

[6] Ravikumar S., Seshagiri V., Pranesh R.V. (2014). Effect of Process Parameters on Mechanical Properties of Friction Stir Welded Dissimilar Materials between AA6061-T651 and AA7075-T651 Alloys. Int. J. Adv. Mech. Eng..

[7] Nandan R., DebRoy T., Bhadeshia H.K.D.H. (2008). Recent advances in friction-stir welding—Process, weldment structure and properties. Prog. Mater. Sci..

[8] Commin L., Dumont M., Masse J.-E., Barrallier L. (2009). Friction stir welding of AZ31 magnesium alloy rolled sheets: Influence of processing parameters. Acta Mater..

[9] Mishra R.S., Ma Z.Y. (2005). Friction stir welding and processing. Mater. Sci. Eng. R.

[10] Senkara J., Zhang H. (2000). Cracking in spot welding aluminum Alloy AA5754. Weld. J..

[11] Miles M.P., Nelson T.W., Decker B.J. (2004). Formability and strength of friction-stir-welded aluminum sheets. Metall. Mater. Trans. A.

[12] Peel M., Steuwer A., Preuss M., Withers P.J. (2003). Microstructure, mechanical properties and residual stresses as a function of welding speed in aluminum AA5083 friction stir welds. Acta Mater..

[13] Kwon Y.J., Shim S.B., Park D.H. (2009). Friction stir welding of 5052 aluminum alloy plates. Trans. Nonferrous Met. Soc. China.

[14] Jin H., Saimoto S., Ball M., Threadgill P.L. (2001). Characterization of microstructure and texture in friction stir welded joints of 5754 and 5182 aluminum alloy sheets. Mater. Sci. Technol..

[15] Attallah M.M., Davis C.L., Strangwood M. (2007). Influence of base metal microstructure on microstructural development in aluminium based alloy friction stir welds. Sci. Technol. Weld. Join..

[16] Kulekci C., Ik A.S., Kaluc E. (2008). Effects of tool rotation and pin diameter on fatigue properties of friction stir welded lap joints. Int. J. Adv. Manuf. Technol..

[17] Barlas Z., Ozsarac U. (2012). Effects of FSW Parameters on Joint Properties of Al Mg3 Alloy. Weld. J..

[18] Garware M., Kridli G.T., Mallick P.K. (2010). Tensile and fatigue behavior of friction-stir welded tailor-welded blank of aluminum alloy 5754. J. Mater. Eng. Perform..

[19] Schmidt H.B., Hattel J.H. (2008). Thermal modelling of friction stir welding. Scr. Mater..

[20] Sheikh-Ahmad J.Y., Ozturk F., Jarrar F., Evis Z. (2016). Thermal history and microstructure during friction stir welding of Al–Mg alloy. Int. J. Adv. Manuf. Technol..

[21] Hwang Y.M., Kang X.W., Chiou Y.C., Hsu H.H. (2008). Experimental study on temperature distributions within the work piece during friction stir welding of aluminum alloys. Int. J. Mach. Tool Manuf..

[22] Chao Y.J., Qi X., Tang W. (2003). Heat Transfer in Friction Stir Welding—Experimental and Numerical Studies. J. Manuf. Sci. Eng..

[23] Schmidt H., Hattel J., Wert J. (2004). An analytical model for the heatgeneration in friction stir welding. Model. Simul. Mater. Sci..

[24] Serio L.M., Palumbo D., Galietti U., De Filippis L.A.C., Ludovico A.D. (2016). Monitoring of the friction stir welding process by means of thermography. Nondestruct. Test. Eval..

[25] Serio L.M., Palumbo D., Galietti U., De Filippis L.A.C., Ludovico A.D. (2014). Analisi del processo di Friction Stir Welding applicato alla lega AA5754-H111: Comportamento meccanico e termico dei giunti. Riv. Ital. Saldatura.

[26] Serio L.M., Palumbo D., De Filippis L.A.C., Galietti U., Ludovico A.D. (2016). Effect of Friction Stir Process Parameters on the Mechanical and Thermal Behavior of 5754-H111 Aluminum Plates. Materials.

[27] Elatharasan G., Senthil Kumar V.S. (2012). Modelling and optimization of friction stir welding parameters for dissimilar aluminium alloys using RSM. Procedia Eng. Int. Conf. Model. Optim. Comput..

[28] Ramanjaneyulu K., Madhusudhan R.G., Hina G. (2015). Optimization of process parameters of aluminum alloy AA2014-T6 friction stir welds by response surface methodology. Defence Technol. Spec. Issue Mater. Join..

[29] Rajakumar S., Balasubramanian V. (2012). Establishing relationships between mechanical properties of aluminium alloys and optimised friction stir welding process parameters. Mater. Des..

[30] Rambabu G., Balaji Naik D., Venkata Rao C.H., Srinivasa Rao K., Madhusudan G.R. (2015). Optimization of friction stir welding parameters for improved corrosion resistance of AA2219 aluminum alloy joints. Def. Technol..

[31] International Organization for Standardization (2012). UNI EN ISO 25239-5:2012: Saldatura Friction Stir—Alluminio—Parte 5: Requisiti di Qualità e di Ispezione.

[32] International Organization for Standardization (2016). ISO 6892-1:2016: Metallic Materials—Tensile Testing—Part 1: Method of Test at Room Temperature.

[33] International Organization for Standardization (2012). UNI EN ISO 4136:2012: Prove Distruttive Sulle Saldature di Materiali Metallici—Prova di Trazione Trasversale.

[34] International Organization for Standardization (2017). UNI EN ISO 17637:2017: Controllo Non Distruttivo Delle Saldature—Esame Visivo Dei Giunti Saldati per Fusione.

[35] Kallee S.W., Nicholas E.D., Burling P.M. TWI. Application of friction stir welding for the manufacture of aluminium ferries. Proceedings of the 4th International Forum on Aluminium Ships.

[36] Lombard H., Hattingh D.G., Steuwer A., James M.N. (2008). Optimising FSW process parameters to minimise defects and maximise fatigue life in 5083-H321 aluminium alloy. Eng. Fract. Mech..

